# Supplementary material for: Comprehensive Genomic Analysis of SnRK in Rosaceae and Expression Analysis of RoSnRK2 in Response to Abiotic Stress in Rubus occidentalis
Source: Plants (Basel). 2023 Apr 26;12(9):1784. doi: 10.3390/plants12091784 (PMC10181103; doi:10.3390/plants12091784)
Supplement: Supplementary file 1 [file plants-12-01784-s001.zip › Supplementary Figures.pdf]

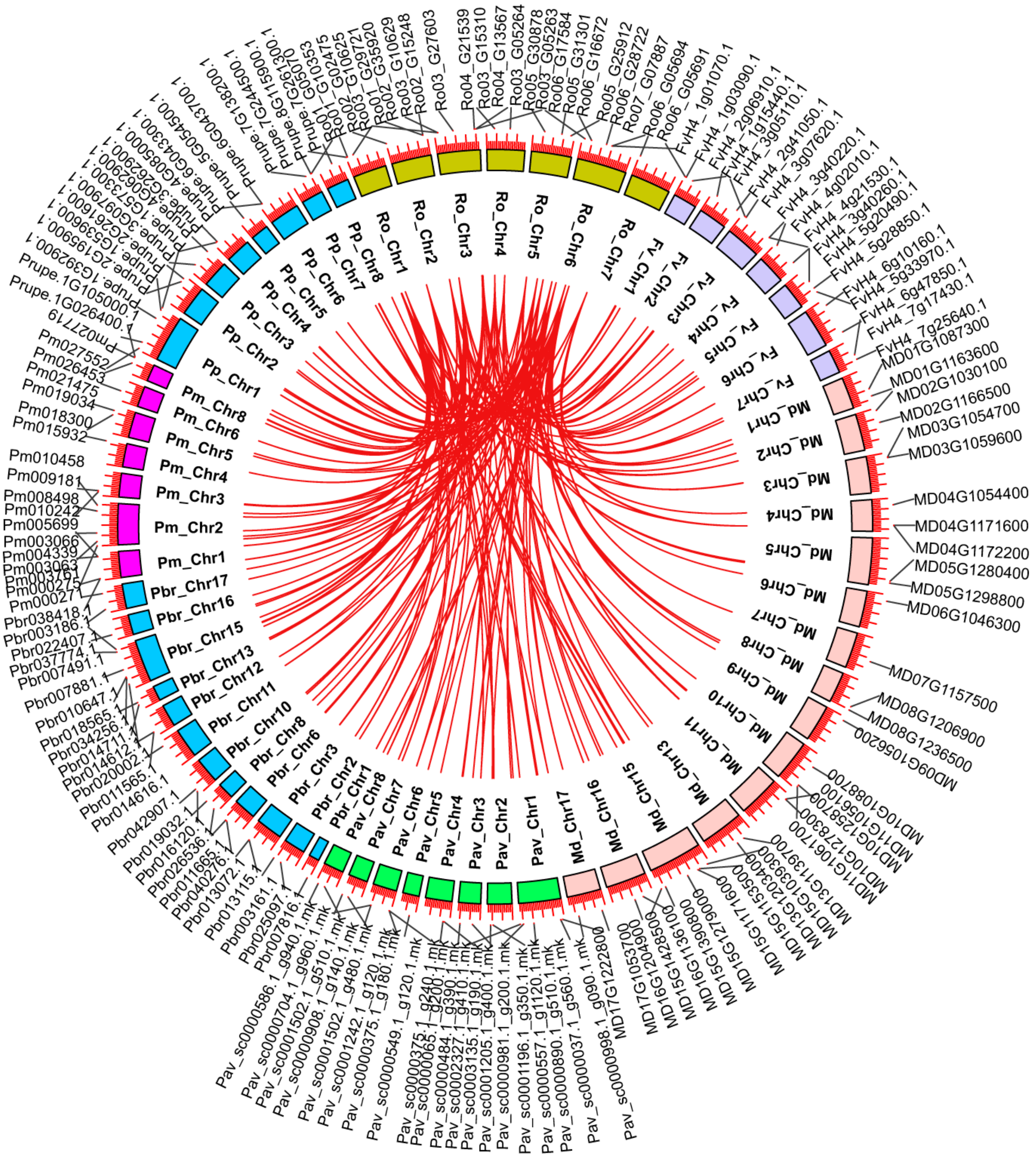

Figure S1. The collinear correlation of the *SnRK* is displayed between black raspberry and six other Rosaceae species.

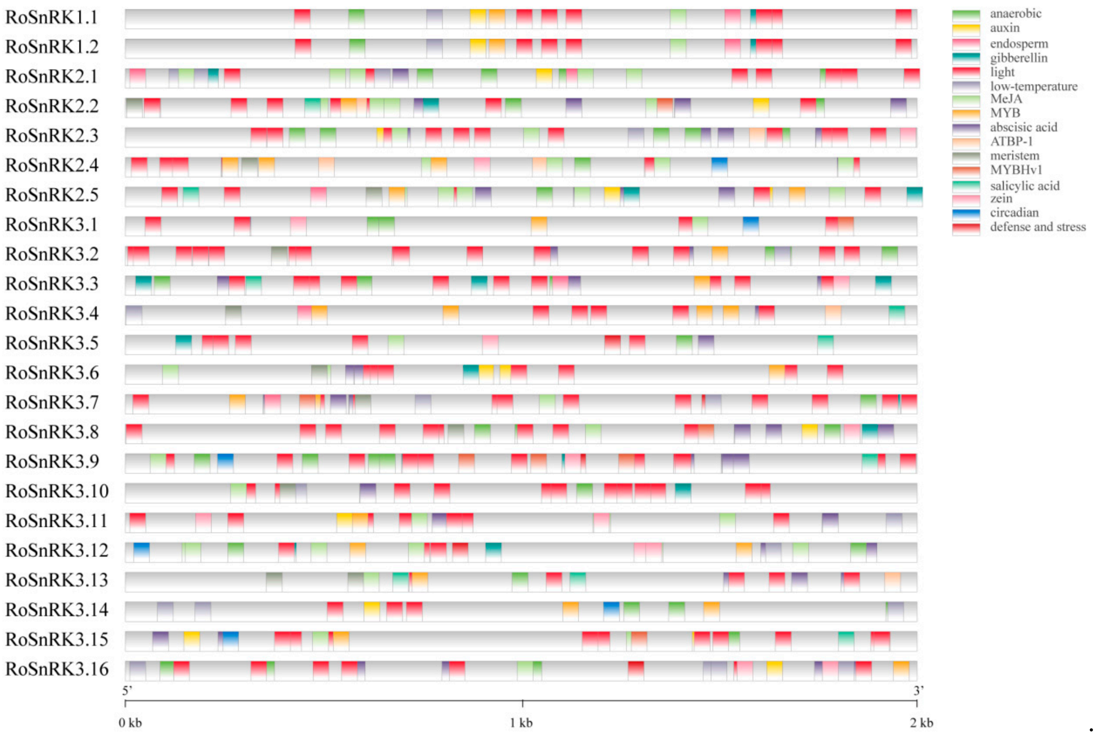

Figure S2. Predicted cis-regulatory elements in *RoSnRK* promoters. Promoter sequences (2000 bp) of 23 *RoSnRK* genes were analyzed by PlantCARE. Boxes with different colours were represented different types of element.
